# Assessment of testicular stiffness in fertile dogs with shear wave elastography techniques: a pilot study

**DOI:** 10.3389/fvets.2024.1397347

**Published:** 2024-05-02

**Authors:** Viola Zappone, Nicola Maria Iannelli, Letizia Sinagra, Giulia Donato, Marco Quartuccio, Santo Cristarella, Massimo De Majo, Tiziana Caspanello

**Affiliations:** ^1^Department of Veterinary Sciences, University of Messina, Messina, Italy; ^2^Clinica Veterinaria Camagna–VetPartners, Reggio di Calabria, Italy

**Keywords:** shear wave elastography, ultrasound, dog, testis, fertility

## Abstract

Ultrasound of the testes is important in the evaluation of breeding dogs, and recently advanced techniques such as Shear Wave Elastography (SWE) have been developed. This study focused on evaluation of normal testicular stiffness in healthy and fertile male dogs, employing both qualitative (2D-SWE) and quantitative (pSWE, 2D-SWE) techniques. Nineteen dogs of various medium-large breeds aged 3.39 ± 2.15 years, and with a history of successful reproduction were included after clinical, B-mode and Doppler ultrasound of testes and prostate, and semen macro and microscopic evaluations. pSWE involved square regions of interest (ROIs) placed at six different points in the testicular parenchyma, while 2D-SWE depicted stiffness with a color scale ranging from blue (soft) to red (stiff), allowing a subsequent quantification of stiffness by the application of 4 round ROIs. The results showed a mean Shear Wave Speed (SWS) of 2.15 ± 0.39 m/s using pSWE, with lower values above the mediastinum compared to below, and in the center of the testis compared to the cranial and caudal poles. 2D-SWE demonstrated a uniform blue pattern in the parenchyma, and a mean SWS of 1.65 ± 0.15 m/s. No significant differences were found between left and right testes, above and below the mediastinum, or among breeds. No correlations were observed between mean SWS and body condition score, age, testicular and prostatic volume. Weight was positively correlated with mean SWS only by 2D-SWE. By performing semen analysis and enrolling only healthy and fertile adult dogs, we ensured both structural and functional integrity of the testes. This pilot study represents a valuable baseline data for testicular stiffness by both pSWE and 2D-SWE with a Mindray US machine in medium-large sized healthy and fertile dogs, pointing out the potential role of SWE in the non-invasive fertility assessment and management of breeding dogs.

## Introduction

The ultrasonographic evaluation of the testes is an important step in assessing the reproductive potential of a male dog, along with clinical examination of the genitalia, assessment of libido and examination of semen (1). Ultrasonography is a reliable imaging technique that allows real-time assessment of the reproductive tract and is the gold standard for testicular evaluation due to its high resolution, ability to assess flow, availability, and safety (2, 3). To improve the accuracy of bidimensional ultrasound, several advanced ultrasound techniques have been developed, such as Doppler, contrast-enhanced ultrasonography (CEUS), 3D/4D ultrasonography, and elastography (3).

Elastography is an ultrasound technique for measuring tissue stiffness. Currently, there are two main categories, Strain elastography (SE) techniques, which measure the elastic recovery of tissue after the application of external mechanical compression with a probe, and Shear wave elastography (SWE) techniques, which are based on the measurement of shear wave speed (SWS). Shear waves are generated in the tissue in response to focused acoustic pulses from the probe, and their speed is positively correlated with tissue stiffness. Moreover, SWE techniques can be divided into point shear-wave elastography (pSWE) and multidimensional SWE (2D-SWE, and 3D-SWE). The former measures the SWS in a focal point in the tissue (called region of interest, ROI), while 2D-SWE measures the SWSs in several points within a larger field of view (FOV) and depicts them in a color-coded map (called elastogram), superimposed to the gray-scale image (4–7). In 3D-SWE, the images are reconstructed in three dimensions (i.e., the axial, sagittal, and coronal planes), using a single sweep of the ultrasound beam across the entire area of interest. Thus, 3D-SWE has the capability to generate volumetric images and to effectively show the stiffest point within a mass (8, 9). Results of SWE can be expressed both in m/s (as shear wave speed) or be converted to Young’s modulus (in kPa) (6).

In human medicine, testicular stiffness has been investigated by elastography in both physiological (10) and pathological testes (11–18). Elastography has been applied for the evaluation of undescended testes, infertility, testicular torsion, tumors, microlithiasis, varicocele and segmental testicular infarction (18), and has shown efficacy in the diagnosis of varicocele (19) and in discriminating focal non-neoplastic lesions from neoplasms, and benign from malignant testicular tumors (20). This technique also showed to be useful in predicting the postoperative improvement in some sperm parameters in patients subjected to varicelectomy (21).

In veterinary medicine, studies on the application of elastography to canine testes have demonstrated its utility in differentiating Leydigomas from non-neoplastic testicular lesions by 2D-SWE (22), in assessing normal testicular values by pSWE (23), in describing pSWE characteristics of pathological testicular tissue (24), and in evaluating epididymal spermatogenesis by SE (25).

The aim of this study was to assess normal testicular stiffness using pSWE and 2D-SWE in dogs with established fertility. This would allow this ultrasound technique to be used as a diagnostic support in course of testicular disorders that may interfere with normal fertility in dogs.

## Materials and methods

### Ethical approval

All treatments, housing and animal care followed EU Directive 2010/63/EU on the protection of animals used for scientific purposes. The Ethics Committee of the Department of Veterinary Medicine and Animal Productions at the University of Messina, Italy (protocol n. 051/2021), approved the protocol and procedures. Informed consent was obtained from each dog owner before its inclusion in the study.

### Animals and inclusion criteria

A total of 19 client-owned healthy male dogs, heterogeneous in breed and age, were included in the study, which took place between April and October 2023. The subjects were presented to a veterinary practice (Clinica Veterinaria Camagna – VetPartners, Reggio Calabria, Italy) for an evaluation of their reproductive potential. To be included in the study, subjects had to be in good general health, have at least one litter in their reproductive history, and have prostatic and testicular echotexture and sizes within the physiological range the animal’s weight and size (26, 27). They also had to be free of clinical and ultrasound abnormalities of the reproductive system and have sperm with minimum characteristics according to the World Health Organization standard methods (28).

For each dog, a remote and recent medical history was acquired, and general clinical examinations and blood analysis were performed. Blood tests included a complete blood count (CBC), and a baseline serum biochemistry assessment of albumin (ALB), globulin (GLOB), ALB/GLOB ratio, alkaline phosphatase (ALKP), Alanine transaminase (ALT), blood urea nitrogen (BUN), creatinine (CREA), glucose (GLU), and total proteins (TP). Finally, a complete reproductive health examination was performed, including a clinical examination of the reproductive system, ultrasound of the testes and prostate, and semen analysis.

### Ultrasound procedures

Ultrasonography was performed by the same operator (MQ), who had 15 years of experience in this field, to avoid interobserver variability. All the procedures on the dogs were performed without sedation or anesthesia. Scrotal sac hair was clipped, and gel was applied to the surface.

B-mode ultrasound was performed by a 3–12 MHz linear transducer (L12-3E) using a Mindray DC-80A ultrasound equipment (Mindray Medical Italy S.R.L. Via Leonardo da Vinci, 158–20,090 Trezzano sul Naviglio, Italia). Qualitative analysis of blood flow by Color and power Doppler was performed in testes, epididymis, and spermatic cord.

Testicular volume was calculated using the electronic calipers of the device, applying the formula for an ellipse: volume = length × width × height × 0.5236. The volume of each testicle was added to give the total testicular volume (TTV). Prostatic volume (PV) was calculated using the formula: [1/2.6 (L × W × D)] + 1.8 (29). Both testes and prostate were assessed for size, margins (regular or irregular) and echotexture of the parenchyma (homogeneous and heterogeneous, hypoechoic, hyperechoic or mixed in relation to the surrounding tissue). Elastography was performed using a linear transducer (L12-3E) to obtain pSWE and 2D-SWE data. To minimize the external pressure of the probe, a thick layer of coupling gel was applied to the scrotal surface and the transducer was gently positioned in order to obtain a longitudinal scan of the testis. The shape of contact surface displayed on the screen was used as a quality criterion for the degree of manual pressure. pSWE was performed using square ROIs of 0.5 × 0.5 cm, positioned in six points of the testicular parenchyma (i.e., cranial, middle, and caudal portions, above and below the mediastinum line, respectively) where there was no vascularization signal. The machine gave real-time median SWS values within the ROI and quality indexes, such as the Motion-stability index (MST-B) and Interquartile range/median (IQR/M) ratio. The first index is represented by a 5-point star-shaped scale that turns green when the object is perfectly still, while the IQR/M ratio is an index of variability of the measurements within the ROI. The criteria for frame acquisition were at least 4 green stars for the MST-B index and an IQR/M ratio < 15% (7, 30). A mean value for each of the six ROI was calculated in m/s.

For the 2D-SWE ultrasound, the FOV was set to include the whole testis in longitudinal scan. When the elastography mode was on, the machine provided a dual image representing the B-mode ultrasound on the left, and the elastogram on the right. The color scale of the elastogram ranged from blue to red (0–70 kPa – 0.0–4.8 m/s), with blue areas representing the softest tissue and red areas representing the stiffest tissue. The criterion for the acquisition of the frames was a MST-B index of at least 4 green stars. Subsequently, in post-processing of the saved image, focal mean SWSs could be assessed by placing round ROIs within the FOV. The operator placed two pairs of ROIs of 0.5 cm diameter on each testis, two ROIs being above the mediastinum line, and two beneath it. Also in this case, the ROIs were positioned in areas without vascularity. Then the average SWSs between the two ROIs of each couple were calculated.

### Semen analysis

Sperm collection was performed in a quiet and appropriate environment with a non-slip floor, by manual collection and in the presence of a teasing bitch, and after removal of the extragonadal reserve to minimize defects in sperm stored in the epididymis, such as reduced motility and increased debris. Macroscopically, the volume, color and appearance of the ejaculate were assessed. Sperm concentration was assessed using an SDM1 photometer (MiniTube™), calibrated for dogs by prior validation with a Makler chamber (Sefi-Medical Instruments, Haifa, Israel), by placing 10 μL of ejaculate in the appropriate loggia of the instrument’s analysis microscope. Motility was assessed using the CASA software. The analysis was performed with the following parameters: number of frames acquired 30, frame rate 60 Hz, minimum cell contrast 75, minimum cell size 4 pixels, straightness threshold 75%, path velocity threshold 100 μm/s-1, mean path velocity (VAP) cut-off 9.0 μm/s-1, mean VAP cut-off 20 μm/s-1, non-motile head size 4 pixels, non-motile head intensity 80, static head size 0.44–4.98, static head intensity 0.49–1.68 and static elongation 17–96%. After staining with eosin/nigrosin, cell morphology was assessed by examining a minimum of 200 spermatozoa per slide. For the evaluation of the semen parameters, we applied the threshold values reported by Tesi et al. (31).

### Statistical analysis

The statistical analysis was performed using software *Jamovi* (Version 2.3.28.0 for MacOS) (32). Descriptive statistics were expressed in mean ± standard deviation, and median values. Shear wave speed (SWS) values were expressed in m/s, in mean values ± standard deviation, 95% confidence interval for the mean, and minimum and maximum values. Shapiro–Wilk test was performed to assess normality of data. Data obtained by 2D-SWE were normally distributed, while data from pSWE did not follow normality; hence, for the analysis of these data sets, parametric and non-parametric tests were employed, respectively.

For pSWE measurements, Mann–Whitney’s U test was performed to evaluate differences between measurements in left and right testicle, and above and below the mediastinum line. Friedman’s test was used for the analysis of variance between the six measurement points. If the test was positive and statistically significant, *post-hoc* Paired Comparisons (Durbin-Conover) were performed.

For 2D-SWE evaluations, Student’s T test was applied to evaluate differences between measurements taken in left and right testis, above and below the mediastinal line.

For both pSWE and 2D-SWE measurements, a Spearman Rho test was applied to search for correlation between of mean SWS with weight, body condition score (BCS), age, testicular volume, and prostatic volume of the dogs. Kruskall-Wallis test was used for the analysis of variance of pSWE and 2D-SWE values between breeds.

For correlations between total testicular volume (TTV) and total sperm count (TSC), TTV and sperm motility percentages, prostatic volume (PV) and TSC, and between ejaculate volume and sperm motility percentages, Rho Spearman’s test was performed.

For all the tests, results with *p* values <0.05 were considered statistically significant.

## Results

### Animals

Nineteen male dogs were included in the study (Table 1), of which seven were English Setters, six Pointers, four Italian Pointing dogs, one German Wirehaired Pointer and one Italian Spinone. The age of the dogs ranged from 1 to 9 years, with a mean of 3.39 ± 2.15 years and a median of 3 years. Their weight ranged from 15.5 to 31 kg, with a mean of 23.19 ± 4.90 kg and a median of 23 kg. Body condition score (BCS) showed a mean score of 4.29 ± 0.75 on a scale of 1 to 9 points. None of the dogs showed alterations of the blood parameters examined. Due to lack of compliance, dogs n.17 and n.18 were only assessed with pSWE and 2D-SWE, respectively.

**Table 1:** Characteristics of subjects enrolled in the study: breed, body weight, body condition score.

Dog	Breed	Body Weight (kg)	Age (Years)	BCS (1–9)
1	Pointer	21	8	4.5
2	Pointer	23	1.5	3.5
3	English Setter	19	9	4
4	Pointer	20	5	4
5	English Setter	18.5	2	3.5
6	English Setter	20	4	4
7	English Setter	24	4	4
8	English Setter	21	4	4
9	Pointer	21	5	5
10	Pointer	23	1.5	4.5
11	Pointer	24	1.5	5
12	Italian Spinone	25	1	6
13	English Setter	15.5	2.5	5
14	English Setter	15.7	2.5	5.5
15	Italian Pointing dog	31	2	4
16	Italian Pointing dog	30	2	4.5
17	Italian Pointing dog	31	3	3.5
18	Italian Pointing dog	30	3	3
19	German Wirehaired Pointer	28	3	4

BCS = Body condition score.

### B-mode and Doppler ultrasound

**Figure 1:**
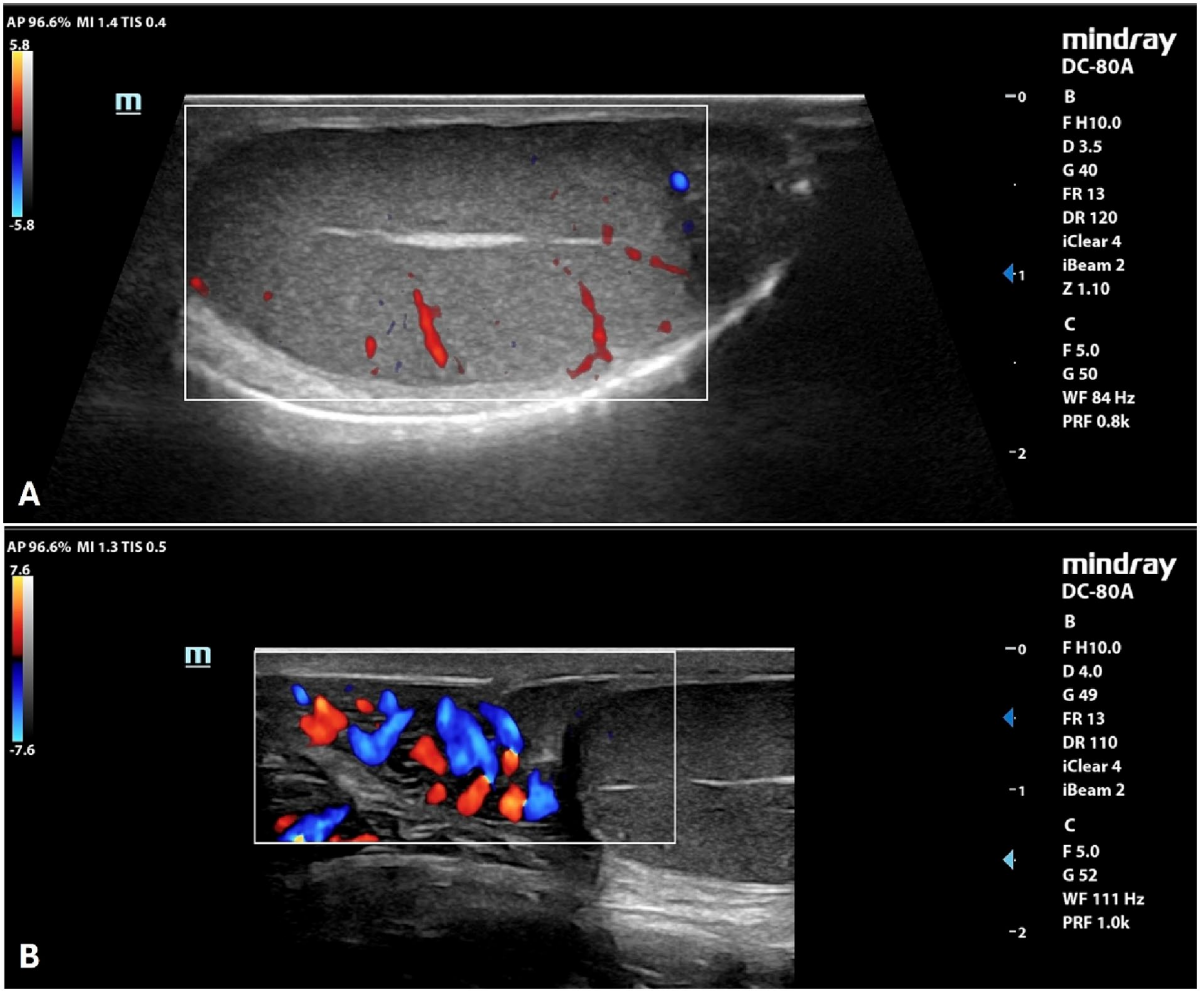
Color Doppler examination of the testicle **(A)** and spermatic cord **(B)** of a normal canine testicle.

By B-mode ultrasound, the testes and the prostate of all the dogs included were normal in size, margins, and parenchyma echotexture. The testes were ovoid in shape, with regular margins characterized by a thin and hyperechoic line (tunica albuginea). Testicular parenchyma presented medium echogenicity with a fine and homogeneous echotexture, interrupted by the presence of the mediastinum in the central part of the testis, which appeared as a hyperechoic line or spot, in the longitudinal and transverse scans, respectively. The prostate showed a rounded shape, with smooth margins and a homogeneous fine-coarse, medium-echoic texture. Color and power-Doppler examinations of testicles, prostate and spermatic cord showed the presence of widely distributed arterial and venous blood flows (Figure 1). Quantitative testicular and prostatic ultrasound evaluations showed a mean total testicular volume (TTV) of 16.62 ± 4.36 cm^3^ and a mean prostatic volume (PV) of 10.88 ± 4.18 cm^3^ (Table 2).

**Table 2:** Total testicular and prostate volume and semen parameters of the ejaculate of the 19 dogs included in the study.

Subjects	Measurements		Quantitative parameters			Motility		
Dog	TTV (cm^3^)	PV (cm^3^)	Volume (ml)	Count (x10^6^/ml)	TSC (x10^6^)	Progr. %	N-Progr. %	Immobile %
1	11.24	14.09	13	53.92	700.96	74	25.46	0.54
2	20.6	11.32	14	38.55	999.04	82	17.89	0.11
3	20.55	8.47	5	133.45	667.25	69.45	27.8	2.75
4	16.42	10.22	5	124.65	623.25	69.19	27.54	3.27
5	12.21	10.9	10	57.84	630.45	59.7	37.72	2.58
6	11.63	5.28	9	161.35	1452.15	28.9	69.85	1.24
7	21.58	6.06	7	22.57	157.99	62.2	26.42	11.38
8	15.73	10.32	3.5	273.18	956.13	65.56	34.44	0
9	10.35	16.54	12	104.03	1248.36	69.36	30.54	0.1
10	20.06	12.68	5	165.76	828.8	74.54	25.46	0
11	19.8	5.6	10	128.17	1281.7	82.77	17.23	0
12	9.23	13.24	4	169.89	679.56	70.63	29.37	0
13	13.77	6.07	9	121.76	1095.84	76.23	23.68	0.09
14	17.05	5.39	8	109.44	875.52	76.61	23.14	0.25
15	21.73	11.78	9	100.33	902.97	71.4	28.83	0.23
16	23.39	20.19	19	87.28	903.42	80.66	19.01	0.33
17	15.61	16.25	20	35.57	1323.36	73	25.82	1.18
18	20.1	12.68	3	36.05	529.98	86	13.85	0.15
19	14.8	9.62	4	36.05	468.65	84	15.93	0.07

US = Ultrasound; TTV = Total testicular volume; PV = Prostatic volume; TSC = Total Sperm count; Progr = Progressive; N-Progr = Non-Progressive.

### pSWE

**Figure 2:**
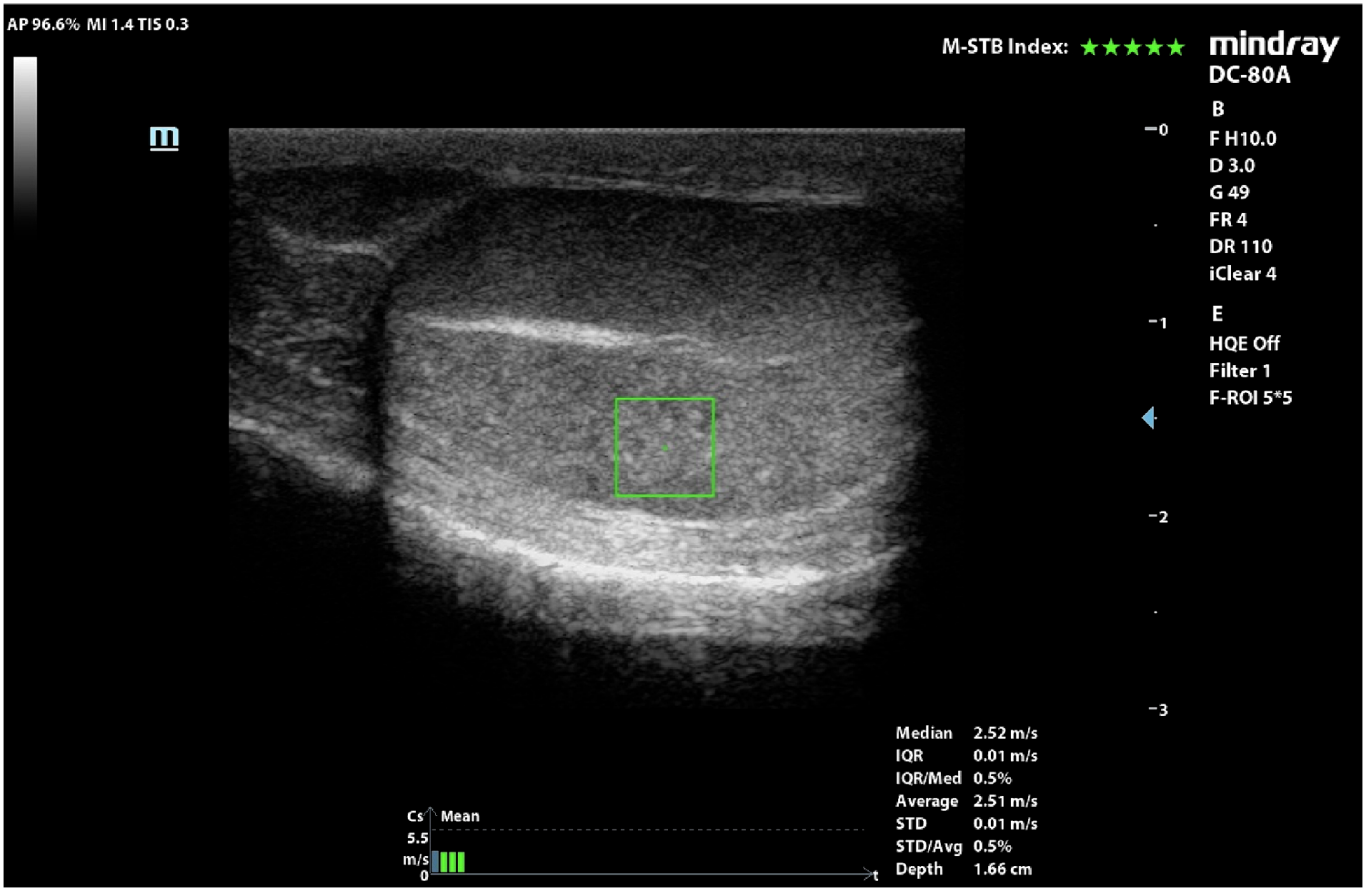
pSWE of a normal dog’s testicle. ROI was positioned in the middle portion and below the mediastinum.

Eighteen pairs of testes were evaluated by pSWE and 216 measurements were obtained (Figure 2). Mean SWS was 2.15 ± 0.39 m/s (95%CI = 2.10–2.21 m/s). Mean ± Standard deviation (SD), 95% confidence interval (CI) for the mean, minimum and maximum SWSs obtained in the six different points of the testes, are reported in Table 3.

**Table 3:** Shear wave speeds (SWSs) obtained by pSWE in the six points of measurement (cranial, middle, and caudal portions of the parenchyma, above and under the mediastinum).

	Above mediastinum			Below mediastinum		
	Cranial	Middle	Caudal	Cranial	Middle	Caudal
Mean ± SD (m/s)	2.15 ± 0.32	1.86 ± 0.23	2.01 ± 0.32	2.41 ± 0.37	2.14 ± 0.44	2.36 ± 0.40
95% CI for the mean (m/s)	2.04–2.26	1.79–1.94	1.9–2.11	2.28–2.53	1.99–2.29	2.22–2.49
Min (m/s)	1.62	1.56	1.42	1.53	1.54	1.51
Max (m/s)	2.81	2.46	3.04	3.07	3.07	3.21

No statistical differences were found between left and right testicles (*p* = 0.189), while the mean values obtained below the mediastinum (2.30 ± 0.41 m/s) were significantly higher than the ones taken above the mediastinum (2.01 ± 0.31 m/s) (*p* < 0.001). Friedman’s test showed significant differences between the measurements taken in the three points of the testicular parenchyma (cranial, middle, caudal portion) (*p* < 0.001). *Post-hoc* comparisons showed that measurements taken in the middle of the testicular parenchyma were significantly lower than measures obtained in the cranial (*p* < 0.001) and caudal (*p* = 0.008) poles. These differences remained when measurements above (*p* = 0.003) and below (*p* < 0.001) the mediastinum were considered separately.

No significant differences were found in mean SWS values between different breeds (*p* = 0.159). No correlation was found between pSWE measurements and weight, BCS, age, testicular volume, prostatic volume (Table 4).

**Table 4:** Analysis of correlation between mean SWS and weight, age, body condition score (BCS), testicular and prostatic volume of the dogs examined by pSWE.

		Weight	Age	BCS	Testicular volume	Prostatic volume
Mean SWS	Spearman’s Rho	0.065	−0.394	0.327	−0.122	0.284
	DoF	16	16	16	16	16
	*p* value	0.797	0.106	0.185	0.631	0.253

BCS, Body condition score; DoF, Degrees of freedom.

### 2D-SWE

Eighteen pairs of testes were evaluated by 2D-SWE and 72 SWS measurements were obtained.

**Figure 3:**
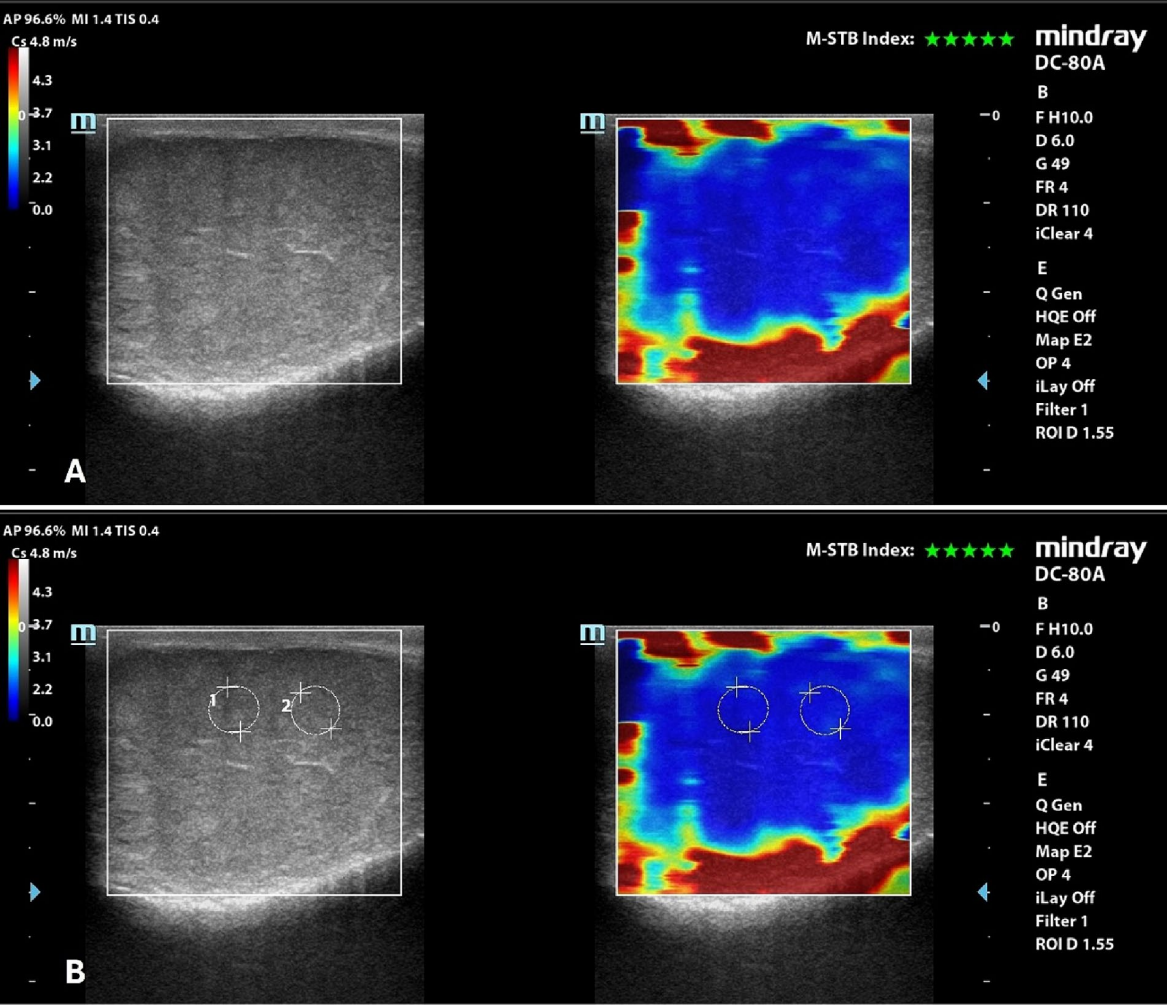
2D-SWE of a normal dog’s testicle, with a dual image showing the B-mode on the left and the elastogram on the right; **(A)** qualitative assessment; **(B)** quantitative assessment obtained by placing 2 round ROIs in the testicular parenchyma above the mediastinum line.

Qualitative analysis showed a uniform blue pattern in the testicular parenchyma, while the surrounding structures (testicular involutes) appeared as a colored border from green to red, following the gradient of the scale, from the internal to the external part of the FOV (Figure 3). Results of quantitative analysis, Mean, with the respective 95% confidence interval (CI), standard deviation (SD), minimum and maximum values, expressed m/s, are reported in Table 5.

**Table 5:** Quantitative values obtained by 2D-SWE of testes.

	SWS (m/s)
Mean ± SD	1.65 ± 0.15
95% CI for the mean	1.62–1.69
Minimum	1.35
Maximum	2.03

No significant differences were found between left and right testicles (*p* = 0.061), and between measurement taken above (1.64 ± 0.15 m/s) and below (1.66 ± 0.15 m/s) the mediastinum line (*p* = 0.486). There were no significant differences also in measurements between breeds (*p* = 0.249).

A significant positive correlation was found between testicular stiffness and weight of dogs. No correlation was found between 2D-SWE values and age, BCS, testicular volume, and prostatic volume of the evaluated patients (Table 6).

**Table 6:** Spearman’s Rho correlation test between mean SWS obtained by 2D-SWE and weight, age, body condition score, total testicular volume, and prostatic volume of the dogs examined.

		Weight	Age	BCS	TTV	PV
mean SWS	Spearman’s Rho	0.498*	−0.17	−0.166	0.034	0.076
	DoF	16	16	16	16	16
	*p* value	0.035	0.5	0.512	0.893	0.764

^*^p < 0.05: DoF = degrees of freedom.

### Semen analysis

The results of the evaluation of the ejaculate of the 19 dogs included in the study are reported in Table 2. The analysis of quantitative parameters showed an average volume of 8.92 ± 4.96 mL of ejaculate, an average concentration of spermatozoa of 103.15 ± 63.26 ×10^6^/ml and a mean total sperm count of 859.23 ± 328.44 ×10^6^ spermatozoa. The evaluation of motility of the spermatozoa showed average percentage values of 71.38 ± 12.57%, 27.37 ± 12.01%, and 1.28 ± 2.66% of progressive, non-progressive, and immobile spermatozoa, respectively. The percentage of spermatozoa with abnormalities was lower than 10%. The values of the semen parameters were in the physiological range for the species, breed and size of each animal, demonstrating their fertility.

Spearman’s Rho test showed absence of correlations between TTV and TSC (*p* = 0.769), between TTV and sperm motility percentages (*p* = 0.656), and between PV and TSC (*p* = 0.542). Ejaculate volume was positively correlated to the percentage of progressive spermatozoa (Rho: 0.498; *p* = 0.035), and negatively correlated to the percentage of non-progressive spermatozoa (Rho: −0.499; *p* = 0.035).

## Discussion

Elastography is a safe and harmless ultrasound technique that allows the depiction of mechanical properties of tissues, and the assessment of tissue stiffness, adding diagnostic power to conventional ultrasound techniques. This technique found important applications in human medicine, being highly predictive of hepatic fibrosis and helpful in the characterization of breast and thyroid nodules (33, 34).

In veterinary medicine, several studies evaluated the performance of elastography in discriminating healthy from abnormal tissue, and in characterizing tissue alterations (35, 36). The application of elastography to canine reproductive tract was reported for the first time in 2015, with the assessment of prostatic strain values in healthy beagle dogs (37); in 2016, SE was used to assess canine testicular, epidydimal, and prostatic physiological strain values (38). Compared to SE techniques, SWE techniques showed higher sensitivity (39, 40) and better inter-operator agreement (41, 42). On the other hand, the repeatability of SWE becomes an issue when comparing measurements obtained from different machines. In fact, ultrasound manufacturers use their own software for elastography, whose measurements differ significantly from each other. For this reason, the guidelines for the application of SWE suggest that each manufacturer should have its own reference values (6, 7, 33). To the best of our knowledge, our is the first study that evaluates the performance of both focal and bidimensional SWE techniques in canine testes with normal semen analysis and in absence of ultrasonographic abnormalities of testes and prostate, using Mindray equipment. Therefore, this work establishes the baseline for standard values, useful for further comparisons with pathological findings.

In our study, dogs that could be considered healthy and fertile were selected to assess their testicular SWS. As the dogs enrolled were breeding dogs, it was not possible to perform cytologic and/or histopathologic evaluation of the testicles to assess their integrity. In fact, these traumatic procedures can cause testicular damage, with complications that may also become evident only in the long term (43, 44). Hence, we applied strict inclusion criteria as evidence of both structural and functional integrity of the testicles; good health and fertility were assessed by the absence of clinical and laboratory alterations, the presence of at least one litter in dogs’ history, and a thorough breeding soundness examination, including B-mode and Doppler ultrasound evaluations and semen analysis.

Although correlations between semen parameters and prostatic and testicular volumes were previously reported (45–47), in our study there were no correlations between testicular volume and total sperm count, between testicular volume and sperm motility, or between prostatic volume and total sperm count. However, this discrepancy could be due to the limited number of dogs evaluated, that could have affected the power of the statistical analysis. Furthermore, ejaculate volume showed correlation with sperm motility, being positively correlated to the percentage of progressive spermatozoa, and negatively correlated to the percentage of immobile spermatozoa. To the best of our knowledge, this is the first report of this finding in dogs. Although it was not the aim of our study, this topic is worth of further investigations involving a lager sample size, to ensure a robust statistical analysis of the factors influencing semen quality.

With pSWE evaluation, by measuring in six points of the testicular parenchyma, we obtained an average SWS of 2.15 ± 0.39 m/s. Noteworthy, we found that measurements taken above the mediastinum showed lower values than the ones taken below, and that the central portion of the parenchyma was softer than the cranial and caudal poles, regardless of the depth of the measurement. Although there is no previous report of this finding in veterinary medicine, it has been previously described in human testes (10, 42), and has been explained as a reflection of the different structural anatomy in the testicular portions examined; in fact, the central part of the testis is characterized by more seminiferous tubules and greater lymphatic and blood vascularization, opposed to a higher tissue density in the rete testis area (42). Considering these differences, it may be practical to assess the stiffness in several points on the testis, which should include at least the central portion and one of the extreme portions (cranial or caudal) of the parenchyma, both above and below the mediastinum. This will allow the operator to perform a thorough and reliable pSWE evaluation, and to avoid biases related to the point of measurement.

In veterinary medicine, the reference values for a healthy testis according to pSWE have been reported in two studies by Feliciano et al. (23, 24). The first was a preliminary study, which found a mean SWS of 1.28 m/s in juvenile dogs, and 1.26 m/s in adult and senior dogs. In that study no semen analysis, nor cytology or histopathology were performed to confirm the healthy status of the testes (23). In the second study, the same authors evaluated both healthy and abnormal testes, histopathology was performed, and an average SWS of 1.30 ± 0.12 m/s for healthy testes was reported (24). Furthermore, the authors described testicular disorders in 36 testes, defining the values for degeneration, atrophy, hypoplasia, orchitis, interstitial cell tumors, Sertoliomas and Leydigomas, and finding that the benign lesions were softer than malignant ones (24). In both their studies, the shear velocities obtained for normal healthy canine testes higher than those observed in humans (0.62–1.01 m/s) (48), probably due to a major presence of fibrous tissue in the testes of dogs compared to those of humans (23, 24). Despite we performed the same acquisition procedure (i.e., six ROIs in the longitudinal scan), higher SWSs than reported by Feliciano et al. were measured (23, 24). Unfortunately, as the authors used an Acuson S2000/Siemens ultrasound machine, we cannot establish whether the nature of this difference is due only to the different ultrasound machines used or there might be other influencing factors.

Glińska-Suchocka et al. (22) evaluated the performance of 2D-SWE in assessing testicular lesions in 9 dogs with non-neoplastic testicular lesions and Leydigomas, reporting that Leydigomas showed a stiffness of 91.85 kPa (corresponding to 5.53 m/s) compared to 11.25 kPa (corresponding to 1.93 m/s) of nonneoplastic lesions. The authors then suggested elastography for the screening of testicular lesions. However, the authors did not include a healthy control group with which to compare our results. Although, also in this case, the ultrasound machine was different than ours, so the value of a comparison would be low (22).

In our study, the 2D-SWE evaluation showed a mean SWS of 1.65 ± 0.15 m/s. SWS by 2D-SWE showed a significant correlation with weight of the dogs. However, the relatively low strength of the correlation, with a borderline *p* value, and the small population sample consisting of medium-large sized dogs, must be taken into account when considering this result. Mean SWSs obtained by pSWE and 2D-SWE differed from each other, the former being higher than the latter. It has previously been reported that these techniques give different values in human testes, since they rely on different sampling techniques, with greater slowing of shear waves in larger FOV/ROIs than in smaller ones (42). Furthermore, 2D-SWE is characterized by better resolution but lower precision at each image pixel than pSWE (5). However, since this kind of comparison has not been made in other studies using Mindray machines, and was not the aim of our study, it should be further assessed with the appropriate accurate analysis.

In a recent study, Gloria et al. (25) performed strain elastography in dogs to assess testicular stiffness in association with seminal parameters of spermatozoa collected from epididymis after orchiectomy, and with histological quantification of fibrous connective tissue. They found that increased testicular stiffness was associated to functional alterations, rather than to connective tissue deposition (25). In human medicine, a correlation of SWS with testicular parenchymal damage and quantitative sperm abnormality was found (49), and elastography has shown good predictive potential for recovery of semen quality after varicocelectomy (21). A similar evaluation with SWE techniques is worth to be done also in dogs. In fact, being SWE quantitative techniques, it would be possible to correlate quantitative parameters of elastography and semen analysis. As our sample was only made of healthy dogs, we could not perform this assessment. A wider sample, including dogs with semen abnormalities, and with different sizes and weights, should be further evaluated; this would provide a thorough characterization of the standard features of testicular SWE, as well as the assessment of the potential predictive value of SWE also for canine semen quality, and perhaps the definition of thresholds values. If a predictive potential for semen quality will be found, SWE could play an essential role as a complementary step to breeding soundness examination in male dogs.

Lack of compliance is one limitation in the use of SWE in animals. In fact, the subject should remain perfectly still during the evaluation in order to obtain the most reliable results (50). This requirement is difficult to achieve in veterinary medicine, unless performing the exam under general anesthesia and with controlled ventilation, to avoid respiratory motion artifacts. Thanks to its extra-abdominal location, SWE of the testes is not affected by respiratory movements. Furthermore, the application of several quality criteria for the acquisition of the images ensured the reliability of our results. Nevertheless, two of the nineteen dogs enrolled became extremely uncompliant and allowed us to perform only half of the examination.

As mentioned above, our is a pilot study whose sample was mainly composed of medium-large sized dogs; thus, the next crucial step is to extend the research also to small, toy and giant sizes, with the same procedures and equipment, in order to create a database of reference values of testicular stiffness in fertile breeding dogs.

## Conclusion

In conclusion, this pilot study provides a valuable baseline data for testicular stiffness using both pSWE and 2D-SWE with a Mindray ultrasound machine in medium-large size dogs with established fertility. The ability to accurately assess testicular health and functionality in a non-invasive manner provides veterinarians with a valuable supplement to their diagnostic approach, contributing to a better reproductive management of canine patients. Further research on a larger sample of dogs of different size are required to fully realize the potential of this innovative approach to improve fertility assessment and management in breeding dogs.

## Data availability statement

The original contributions presented in the study are included in the article/supplementary material, further inquiries can be directed to the corresponding authors.

## Ethics statement

The animal studies were approved by University of Messina, Italy (protocol n. 051/2021). The studies were conducted in accordance with the local legislation and institutional requirements. Written informed consent was obtained from the owners for the participation of their animals in this study.

## Author contributions

VZ: Methodology, Writing – original draft, Writing – review & editing. NMI: Resources, Supervision, Validation, Writing – review & editing. LS: Investigation, Methodology, Writing – original draft. GD: Formal analysis, Writing – review & editing. MQ: Conceptualization, Methodology, Supervision, Writing – review & editing. SC: Supervision, Validation, Writing – review & editing. MDM: Conceptualization, Methodology, Writing – review & editing. TC: Conceptualization, Methodology, Writing – original draft.
